# Altered Lipid Metabolism in Psoriatic Arthritis: A Comprehensive Review

**DOI:** 10.3390/metabo16050287

**Published:** 2026-04-22

**Authors:** Stanislava Popova-Belova, Mariela Geneva-Popova, Stefka Stoilova, Velichka Popova, Georgi Nikolov, Dimitar Nikolov

**Affiliations:** 1 Department of Propedeutics of Internal Diseases, Faculty of Medicine, Clinic of Rheumatology, University Hospital “Sv. George”, Medical University—Plovdiv, 4002 Plovdiv, Bulgaria; spopova92@abv.bg; 2 University Hospital “Sv. George”, Medical University—Plovdiv, 4002 Plovdiv, Bulgaria; ef_i@abv.bg; 3 Department of Propedeutics of Internal Diseases “Prof. Anton Mitov”, Faculty of Medicine, Clinic of Rheumatology, University Hospital “Kaspela”, Medical University—Plovdiv, 4002 Plovdiv, Bulgaria; drvpopova@gmail.com; 4 Second Department of Internal Diseases, Faculty of Medicine, Clinic of Nephrology, University Hospital “Sv. George”, Medical University—Plovdiv, 4002 Plovdiv, Bulgaria; georgi.nikolov@mu-plovdiv.bg (G.N.); dimitar.nikolov@mu-plovdiv.bg (D.N.)

**Keywords:** psoriatic arthritis, lipid metabolism, dyslipidemia, adipokines, cardiovascular risk, metabolic syndrome, inflammation

## Abstract

Psoriatic arthritis (PsA) is a chronic inflammatory disorder affecting both the joints and skin. Beyond musculoskeletal manifestations, patients with PsA frequently exhibit alterations in lipid metabolism, contributing to an increased risk of cardiovascular disease. Dyslipidemia in PsA arises from multiple mechanisms, including systemic inflammation, insulin resistance, and imbalances in adipokines such as leptin, adiponectin, and resistin. A structured literature search was conducted in PubMed, Scopus, and Web of Science to identify relevant studies on lipid metabolism in psoriatic arthritis, and the evidence was synthesized narratively. PsA is also commonly associated with obesity and metabolic syndrome, further exacerbating dyslipidemia and cardiovascular risk. Interventions including weight loss, lifestyle modification, and anti-inflammatory treatments have been shown to improve lipid profiles and clinical outcomes. This review provides a comprehensive overview of current knowledge on altered lipid metabolism in PsA, highlighting underlying mechanisms, clinical implications, and therapeutic strategies to reduce cardiovascular risk.

## 1. Introduction

Psoriatic arthritis (PsA) is a chronic immune-mediated inflammatory disorder characterized by a heterogeneous spectrum of musculoskeletal and dermatologic manifestations, including peripheral arthritis, enthesitis, dactylitis, axial involvement, and psoriatic skin lesions [1,2]. Approximately 20–30% of patients with psoriasis develop PsA during the course of their disease, highlighting the close pathogenetic relationship between cutaneous and joint inflammation. Beyond its classical clinical features, PsA is increasingly recognized as a systemic disease with metabolic consequences, particularly in lipid metabolism and cardiovascular risk [3].

Dyslipidemia in PsA is multifactorial, resulting from the interplay between chronic systemic inflammation, adipokine dysregulation, insulin resistance, and genetic susceptibility [4,5,6,7].

Pietrzak et al. [1] and Wang et al. [2] reported significant alterations in serum lipid profiles of PsA patients compared to healthy controls and psoriasis patients without arthritis, including reduced high-density lipoprotein (HDL), elevated low-density lipoprotein (LDL), triglycerides, and a higher apolipoprotein A1/apolipoprotein B (ApoB/ApoA1) ratio. Ishchenko et al. [4] demonstrated that even treatment-naïve early PsA patients exhibit low ApoA1 and high ApoB levels, emphasizing the primary role of the disease itself in driving lipid disturbances. Wójcik et al. [8] found differential changes in lipid metabolism in peripheral blood mononuclear cells of PsA patients, indicating that cellular lipid handling is also affected.

Adipokines play a critical role in linking adiposity, inflammation, and lipid metabolism in PsA. Leptin and resistin, generally elevated in PsA, promote pro-inflammatory pathways and are associated with disease activity, whereas adiponectin, which exerts anti-inflammatory and insulin-sensitizing effects, is often reduced [5,6,7,9,10,11,12,13,14,15,16,17,18]. These adipokine alterations are not merely markers but active modulators of lipid homeostasis, insulin sensitivity, and endothelial function, contributing to an atherogenic profile [6,14,18,19,20]. Caso et al. [5] highlighted the pro-inflammatory adipokine profile in PsA, particularly in patients with pronounced skin involvement, while Landgren et al. [18] further linked adipokine levels to both metabolic syndrome components and disease activity scores.

However, most of these studies are cross-sectional and cannot establish causality between adipokine alterations and disease activity. In addition, the reported associations are not entirely consistent across different patient populations, suggesting that adipokine profiles may be influenced by confounding factors such as obesity, treatment status, and disease duration [21,22,23].

Metabolic syndrome, obesity, and insulin resistance are highly prevalent in PsA, more so than in psoriasis without arthritis and the general population [15,22,23,24,25,26,27]. Leite et al. [9] observed that higher body adiposity, fat intake, and cholesterol serum levels correlate with increased disease activity, suggesting a bidirectional relationship between metabolic dysfunction and PsA severity. Haroon et al. [28] reported that metabolic syndrome is associated with more severe disease phenotypes, emphasizing the clinical relevance of identifying and managing metabolic comorbidities [3,28,29,30,31,32,33,34]. Nevertheless, it remains challenging to determine whether metabolic syndrome is a driver of disease severity or a consequence of chronic inflammation and reduced physical activity. The bidirectional nature of this relationship is still debated [28,35,36,37,38,39,40,41,42,43].

Cardiovascular disease represents a major long-term risk in PsA patients [28,35,36,37,38,39,40,41,42,43]. Subclinical atherosclerosis, detected through carotid ultrasound or coronary imaging, is frequently present even in early or minimally symptomatic patients, independent of traditional cardiovascular risk factors [21,31,44,45,46,47,48,49,50,51,52,53]. Szentpetery et al. [53] demonstrated that higher coronary plaque burden in PsA patients is associated with disease severity, independent of metabolic syndrome, highlighting the impact of chronic systemic inflammation. Eder et al. [37] confirmed that increased inflammatory burden correlates with the extent of atherosclerotic plaques, reinforcing the concept that PsA-driven dyslipidemia contributes to accelerated atherogenesis.

Therapeutic interventions targeting both inflammation and metabolic dysfunction can favorably influence lipid metabolism and clinical outcomes. Weight loss through lifestyle modification or bariatric interventions improves response to Tumor Necrosis Factor (TNF) inhibitors and increases the likelihood of achieving minimal disease activity [32,33,34,54,55,56,57,58]. Biologic and conventional Disease-modifying antirheumatic drugs (DMARDs) therapies, including methotrexate and TNF inhibitors, may modulate lipid profiles, though the effects are heterogeneous and partially dependent on baseline metabolic status [2,52]. These findings underscore the importance of a comprehensive, multidisciplinary approach in managing PsA, addressing both musculoskeletal and metabolic components [59,60,61,62,63,64,65,66,67,68,69,70,71,72].

Across individual studies, PsA has generally been associated with lower HDL levels and higher LDL, triglyceride, ApoB, and ApoB/ApoA1 ratios compared with healthy controls and, in some studies, compared with psoriasis without arthritis [1,2,4,8,9]. However, these findings should be interpreted with caution because the available studies differ substantially in sample size, patient characteristics, treatment exposure, and laboratory methodology. Similarly, most studies report increased leptin and resistin levels and reduced adiponectin in PsA, but the magnitude of these alterations and their associations with disease activity vary across cohorts [5,17,18,20]. Therefore, the available evidence is more appropriately summarized narratively than as directly comparable pooled values.

Despite growing evidence, gaps remain in our understanding of the precise mechanisms linking inflammation, adipokine dysregulation, insulin resistance, and lipid abnormalities in PsA. Moreover, the interplay between genetic predisposition and environmental factors, including diet and physical activity, requires further elucidation [29,30,43].

This review aims to integrate current knowledge on altered lipid metabolism in PsA, providing a framework for mechanistic understanding, clinical implications, and therapeutic strategies to mitigate cardiovascular risk.

## 2. Methods

A literature search was performed in PubMed, Scopus, and Web of Science to identify relevant publications on altered lipid metabolism in psoriatic arthritis. The search included articles published up to January 2026 using combinations of the terms “psoriatic arthritis”, “lipid metabolism”, “dyslipidemia”, “apolipoproteins”, “adipokines”, “metabolic syndrome”, “insulin resistance”, and “cardiovascular risk”. Only English-language publications were considered. Original articles, observational studies, mechanistic studies, systematic reviews, and narrative reviews relevant to the topic were included. Reference lists of selected papers were also manually screened for additional eligible studies. Because of substantial heterogeneity among study designs, populations, and reported outcomes, the evidence was synthesized narratively and no formal meta-analysis was undertaken.

### 2.1. Clinical Implications and Cardiovascular Risk in Psoriatic Arthritis

#### 2.1.1. Elevated Cardiovascular Risk in PsA

PsA patients exhibit a markedly increased risk of cardiovascular (CV) morbidity and mortality compared to the general population and even compared to patients with psoriasis alone [10,16,50]. Epidemiological studies have demonstrated higher rates of myocardial infarction, stroke, and coronary artery disease in PsA, which are not fully explained by traditional risk factors alone [38,39,50]. Although these associations are well documented, most available data are derived from observational studies, and residual confounding by traditional cardiovascular risk factors cannot be fully excluded. Therefore, the independent contribution of PsA-related inflammation to cardiovascular risk remains an area of ongoing investigation.

Eder et al. [10] emphasized that chronic systemic inflammation, metabolic disturbances, and dyslipidemia synergistically elevate cardiovascular risk in PsA patients.

Subclinical atherosclerosis is common in PsA, even among patients with minimal joint symptoms or early disease. Carotid ultrasound studies reveal increased intima-media thickness (IMT) and higher prevalence of carotid plaques, correlating with systemic inflammation markers and disease activity [21,31,38,62]. Szentpetery et al. [53] reported that PsA patients exhibit higher coronary plaque burden than matched controls, independent of metabolic syndrome, highlighting the critical role of inflammation-driven atherogenesis. Cooksey et al. [41] confirmed that both carotid and coronary vascular changes are present in PsA, linking lipid dysregulation with structural vascular alterations.

**Figure 1:**
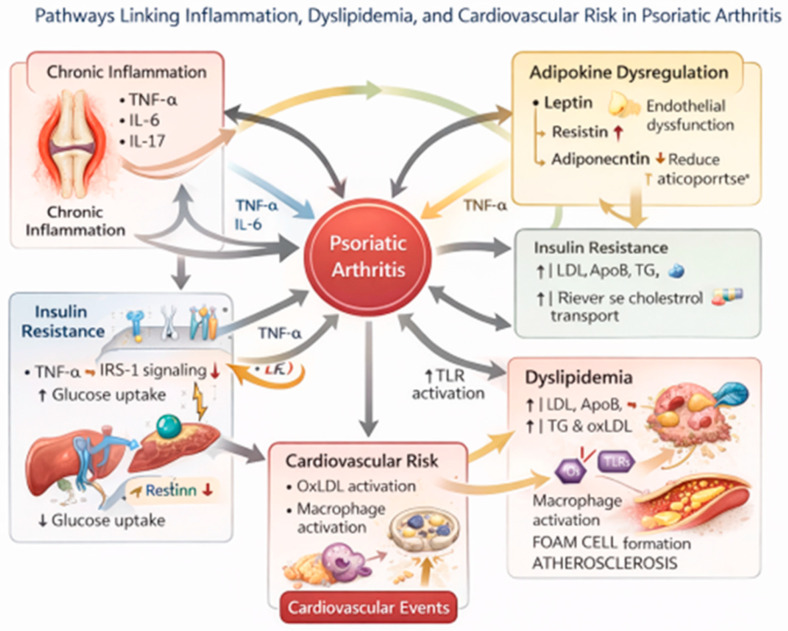
Mechanistic pathways linking inflammation, adipokine dysregulation, insulin resistance, and dyslipidemia in psoriatic arthritis and their contribution to cardiovascular risk. Pro-inflammatory cytokines such as TNF-α, IL-6, and IL-17 suppress lipoprotein lipase activity, enhance hepatic VLDL production, and impair insulin signaling via IRS-1 pathways. Adipokines, including increased leptin and resistin and decreased adiponectin, further promote insulin resistance and endothelial dysfunction. Dyslipidemia, characterized by elevated LDL, triglycerides, and oxidized LDL, activates macrophages through Toll-like receptor pathways, leading to foam cell formation, atherosclerosis, and cardiovascular events. Bidirectional interactions between inflammation, metabolic dysfunction, and lipid abnormalities are illustrated.

Figure 1 illustrates the proposed pathway linking PsA and dyslipidemia, as well as the main therapeutic strategies in PsA.

#### 2.1.2. Metabolic Syndrome and Insulin Resistance

Metabolic syndrome is highly prevalent in PsA and significantly contributes to cardiovascular risk. Maharaj et al. [22] found that the risk of metabolic syndrome in PsA is higher than in psoriasis alone and the general population. Haroon et al. [28] and Wu et al. [35] demonstrated that metabolic syndrome is associated with more severe joint and skin disease, suggesting a bidirectional relationship between metabolic dysfunction and PsA activity. Components of metabolic syndrome, including central obesity, hypertriglyceridemia, low HDL cholesterol, and insulin resistance, exacerbate the pro-atherogenic lipid profile in PsA [9,46,55,57].

Insulin resistance impairs lipid handling and promotes endothelial dysfunction. Reduced lipoprotein lipase activity, impaired reverse cholesterol transport, and elevated circulating free fatty acids increase triglyceride-rich lipoproteins and oxidized LDL, which drive foam cell formation and early atherogenesis [3,29,36]. Yuliasih et al. [46] demonstrated that PsA patients exhibit elevated HOMA-IR scores and impaired beta-cell function, confirming insulin resistance as a central mediator linking inflammation, dyslipidemia, and CV risk.

#### 2.1.3. Role of Adipokines in Cardiovascular Risk

Adipokine dysregulation in PsA contributes to systemic inflammation, insulin resistance, and vascular pathology. Elevated leptin and resistin levels, along with reduced adiponectin, are associated with endothelial activation, pro-thrombotic states, and adverse lipid profiles [5,17,18,20]. Eder et al. [17] demonstrated that serum leptin correlates with both disease activity and subclinical atherosclerosis, whereas low adiponectin levels are associated with higher carotid IMT and plaque burden. Su X, Peng [3] highlighted that adipokines function as both biomarkers and mediators of cardiovascular risk, emphasizing the need for targeted metabolic interventions.

#### 2.1.4. Clinical Studies Linking Lipid Alterations and CV Events

Several cohort and case–control studies have confirmed the association between dyslipidemia in PsA and cardiovascular outcomes. Leung et al. [12] observed that low HDL and high LDL, triglycerides, and ApoB levels correlate with increased incidence of cardiovascular events. Di Minno et al. [19] performed a systematic review and meta-analysis confirming that markers of dyslipidemia, such as elevated ApoB/ApoA1 ratios and triglycerides, are predictive of cardiovascular events in PsA.

Prediabetic metabolic profiles, including insulin resistance and hypertriglyceridemia, have been linked to higher CV morbidity [13,54]. Agca et al. [15] reported that prediabetic PsA patients exhibited increased rates of myocardial infarction and stroke over longitudinal follow-up, emphasizing the clinical relevance of early metabolic screening.

#### 2.1.5. Therapeutic Interventions and CV Risk Modulation

Therapies targeting both inflammation and metabolic dysfunction can reduce cardiovascular risk in PsA. Weight loss and lifestyle interventions improve lipid profiles, insulin sensitivity, and disease activity. Landgren et al., Klingberg et al. [32,56] demonstrated that intentional weight loss in obese PsA patients improves disease activity scores and decreases pro-atherogenic lipid markers. Di Minno et al. [19] further showed that weight reduction enhances the efficacy of TNF inhibitors and increases rates of minimal disease activity achievement (Table 1).

However, heterogeneity among included studies, including differences in study design, patient populations, and lipid measurements, limits the generalizability of these findings. Standardized prospective studies are needed to better define the predictive value of lipid alterations in PsA.

DMARDs and biologics modulate systemic inflammation and partially normalize lipid metabolism. Methotrexate has been associated with improved ApoB/ApoA1 ratios and reduced cardiovascular risk [2]. TNF inhibitors reduce systemic inflammation, improve endothelial function, and may favorably alter lipid profiles [33,34,52,57,63]. Nevertheless, the effects are heterogeneous and influenced by baseline metabolic status and coexisting obesity [25,40,52,70].

Statin therapy, as an adjunct to anti-inflammatory treatment, further mitigates cardiovascular risk by lowering LDL and stabilizing atherosclerotic plaques. Eder et al. [37] suggested that a combination of anti-inflammatory therapy, lipid-lowering agents, and lifestyle modification is optimal for reducing long-term cardiovascular morbidity in PsA

Integrated Management and Clinical Recommendations

Given the multifactorial pathogenesis of cardiovascular risk in PsA, a comprehensive management strategy is essential:

Routine cardiovascular risk assessment includes lipid profiles, blood pressure, BMI, and metabolic syndrome screening [10,23,50].

To reduce cardiovascular risk, it is additionally necessary to implement:

A. Inflammation control: The use of disease-modifying antirheumatic drugs (DMARDs) and biologic agents is aimed at reducing systemic inflammation and the associated disturbances in lipid metabolism [2,33,52].
B. Lifestyle modification: Weight reduction, increased physical activity, and dietary optimization are recommended to improve insulin sensitivity and lipid profile [32,34,56].
C. Adjunctive pharmacotherapy: The use of statins and antihypertensive agents should be considered based on the individual cardiovascular risk profile of the patient [10,23,50].
D. Monitoring of subclinical atherosclerosis: Carotid ultrasound or coronary imaging may be useful in high-risk patients to guide early therapeutic intervention [21,31,53].

Available studies emphasize that early identification and appropriate management of dyslipidemia and metabolic syndrome in patients with PsA not only reduce cardiovascular risk but may also contribute to improved clinical outcomes [9,32,34,40].

Summary

Psoriatic arthritis is associated with significant cardiovascular risk driven by chronic systemic inflammation, adipokine dysregulation, insulin resistance, and pro-atherogenic lipid profilesDyslipidemia in PsA is characterized by low HDL, high LDL and triglycerides, and an elevated ApoB/ApoA1 ratio, which contributes to accelerated atherogenesis. Subclinical atherosclerosis is prevalent even in early disease, underscoring the need for proactive cardiovascular assessment. Integrative management combining anti-inflammatory therapy, metabolic optimization, weight loss, and pharmacologic lipid-lowering interventions is essential for reducing long-term cardiovascular morbidity and improving overall patient outcomes [10,21,53,56,58,67].

### 2.2. Therapeutic Strategies and Lipid Modulation in Psoriatic Arthritis

#### 2.2.1. Conventional DMARDs and Lipid Profiles

Conventional disease-modifying antirheumatic drugs (cDMARDs), such as methotrexate, leflunomide, and sulfasalazine, remain cornerstone therapies in PsA management [59]. Beyond their anti-inflammatory effects, these agents can influence lipid metabolism. Wang et al. [2] demonstrated that methotrexate treatment in PsA patients reduced the ApoB/ApoA1 ratio, indicating a shift toward a less atherogenic lipid profile, particularly in male patients. Methotrexate may exert these effects by decreasing systemic inflammation, which in turn reduces hepatic VLDL synthesis and improves HDL functionality [24].

However, the impact of cDMARDs on lipids is variable. While some studies report modest improvements in HDL and reductions in inflammatory lipoproteins, others show minimal effect, suggesting that lipid modulation is partially dependent on baseline metabolic status and disease activity [4,12,26,36].

#### 2.2.2. Biologic DMARDs and Lipid Modulation

Biologic therapies targeting specific inflammatory pathways have demonstrated profound effects on both PsA disease activity and lipid metabolism [60].

##### TNF-α Inhibitors

TNF inhibitors (e.g., etanercept, adalimumab, infliximab) reduce systemic inflammation and improve vascular endothelial function. Multiple studies show that TNF blockade is associated with:

Increased HDL cholesterol levels

Stabilization or modest reduction in LDL and triglycerides

Decreased ApoB/ApoA1 ratios

Reduction in subclinical atherosclerosis progression [33,34,52,57].

Despite these findings, the effects of TNF inhibitors on lipid profiles are heterogeneous, and improvements in lipid parameters do not always translate into reduced cardiovascular events. This suggests that inflammation reduction alone may not fully normalize cardiovascular risk.

Di Minno et al. [19] highlighted that weight loss combined with TNF inhibitor therapy significantly enhances minimal disease activity achievement and further improves lipid parameters. Leung et al. [12] corroborated these findings, showing that TNF inhibitors reduce inflammation-driven dyslipidemia, particularly in patients with baseline metabolic syndrome.

##### IL-17 and IL-23 Blockers

Emerging evidence suggests that IL-17 and IL-23 inhibitors may influence cardiovascular risk through reduction in systemic inflammation. However, direct evidence demonstrating their effects on lipid profiles or HDL functionality remains limited. Most available studies focus on inflammatory and adipokine pathways rather than direct lipid modulation. Therefore, any potential beneficial effects on lipid metabolism and subclinical atherosclerosis should be interpreted with caution and require further investigation in dedicated clinical studies.

##### Lifestyle Interventions

Lifestyle modification, including weight reduction, physical activity, and dietary optimization, is a critical component of PsA management with dual benefits for disease activity and lipid metabolism [69].

##### Weight Loss

Obesity is strongly associated with more severe PsA, higher disease activity, reduced treatment response, and pro-atherogenic lipid profiles [25,32,40,52,56,72]. Klingberg et al. [32] demonstrated that intentional weight loss improves disease activity scores, reduces leptin levels, increases adiponectin, and improves HDL cholesterol. Di Minno et al. [34] confirmed that weight reduction enhances the efficacy of TNF inhibitors and improves cardiovascular risk markers.

##### Physical Activity and Diet

Regular exercise improves insulin sensitivity, reduces triglycerides, and enhances HDL levels, while dietary interventions—such as reduced saturated fat and increased omega-3 fatty acids—can attenuate systemic inflammation and improve lipid profiles [9,43]. Leite et al. [9] found that higher fat intake correlated with increased PsA activity, suggesting that nutritional counseling is a valuable adjunct to pharmacologic therapy.

##### Pharmacologic Lipid-Lowering Therapies

Statins are the mainstay for pharmacologic management of dyslipidemia in PsA. They are:

Lower LDL and total cholesterol

Stabilize atherosclerotic plaques

Exhibit anti-inflammatory properties that may complement DMARD therapy [10,23,50].

Early initiation of statins in patients with PsA and elevated CV risk markers, particularly in combination with DMARDs and lifestyle interventions, provides additive cardiovascular protection [21,53,71,72].

Integrative Therapeutic Approach

Optimal management of PsA requires a multifactorial, personalized approach targeting inflammation, metabolic dysfunction, and cardiovascular risk simultaneously:

Anti-inflammatory therapy: DMARDs and biologics to control disease activity and reduce cytokine-mediated lipid alterations [2,33,52,57].

Lifestyle modification: Weight loss, exercise, and dietary interventions to improve insulin sensitivity, adipokine balance, and lipid profile [9,24,32,56].

Lipid-lowering pharmacotherapy: Statins for patients with persistent dyslipidemia or high cardiovascular risk [10,23,50].

Monitoring: Regular assessment of lipid profiles, metabolic syndrome components, and subclinical atherosclerosis [21,31,53].

Evidence suggests that combination strategies not only reduce cardiovascular risk but also enhance disease outcomes, including rates of minimal disease activity, remission, and treatment response [32,34]. Personalized therapeutic plans should consider baseline metabolic status, disease phenotype, and comorbidities to maximize both rheumatologic and cardiometabolic benefits (Table 2).

Summary

Therapeutic strategies in PsA extend beyond joint and skin inflammation control to encompass metabolic and cardiovascular protection. Conventional DMARDs, biologic therapies, weight reduction, lifestyle modification, and statins collectively influence lipid metabolism, insulin sensitivity, and adipokine balance. Evidence from interventional and observational studies demonstrates that integrating pharmacologic and lifestyle approaches improves both disease activity and long-term cardiovascular outcomes [2,9,32,33,34,52,56,58].

Future Perspectives and Research Directions in Psoriatic Arthritis and Lipid Metabolism

Emerging Biologic and Small Molecule Therapies

Recent advances in the understanding of PsA pathogenesis have led to the development of targeted biologics and small molecule inhibitors that may influence both inflammation and lipid metabolism [41,47].

JAK Inhibitors and Intracellular Signaling Modulators

Janus kinase (JAK) inhibitors, including tofacitinib and upadacitinib, modulate multiple pro-inflammatory cytokine pathways. By reducing IL-6, TNF-α, and interferon-mediated signaling, JAK inhibitors may indirectly improve lipid profiles and vascular endothelial function [10,20,23]. Early studies in inflammatory arthritis indicate increases in HDL and favorable ApoB/ApoA1 ratios post-treatment, although the long-term cardiovascular benefits require further evaluation [24,26,36].

Importantly, JAK inhibitors have also been associated with increases in LDL cholesterol, and their overall cardiovascular safety profile remains under debate. Data from large clinical trials in inflammatory arthritis have raised concerns regarding major adverse cardiovascular events, underscoring the need for cautious interpretation.

Novel Biologics

Newer biologics targeting IL-17A/F, IL-23p19, and GM-CSF may offer dual benefits: suppression of joint and skin inflammation and modulation of lipid and metabolic pathways. IL-17 blockade has shown promise in improving HDL functionality and reducing vascular inflammation, while IL-23 inhibitors may attenuate adipokine-mediated dyslipidemia [5,17,20,23].

Potential Direct Lipid-Modifying Agents

Experimental approaches aiming to directly normalize lipid metabolism, including ApoA1 mimetics, CETP inhibitors, and PCSK9 inhibitors, represent promising adjuncts in PsA. While primarily studied in general cardiovascular populations, these agents could complement anti-inflammatory therapy to reduce atherosclerotic risk in PsA [10,50,53].

Integrative Biomarker and Imaging Strategies

The future of PsA management lies in personalized medicine guided by precise biomarker and imaging assessments.

Lipidomics and metabolomics: Detailed profiling of circulating lipids and metabolites may identify patient-specific dyslipidemic patterns linked to inflammation, enabling tailored therapy [4,8,29,36].

Adipokine profiling: Measuring leptin, adiponectin, resistin, and visfatin levels could refine cardiovascular risk stratification and guide therapeutic interventions [5,17,18,20].

Advanced imaging: Coronary CT, carotid ultrasound, and PET-CT imaging can detect subclinical atherosclerosis and monitor response to therapy [21,31,53].

These strategies will allow clinicians to identify high-risk patients early and intervene with combined anti-inflammatory, lifestyle, and lipid-targeted therapies [54].

Personalized and Integrated Treatment Approaches

Future research should focus on multimodal interventions combining:

Targeted biologics or small molecules to reduce systemic inflammation [2,33,52,57].

Lifestyle interventions to correct obesity, insulin resistance, and diet-related dyslipidemia [9,32,34,56].

Pharmacologic lipid-lowering therapies for residual atherogenic risk [10,23,50].

Precision medicine approaches guided by lipidomics, adipokine profiles, and imaging biomarkers [4,5,8,17,21].

Integrating these approaches may improve long-term cardiovascular outcomes while enhancing joint and skin disease control.

Research Gaps and Future Directions

Despite advances, several key questions remain:

Mechanistic links between inflammation and lipid dysregulation: Although chronic inflammation is known to induce pro-atherogenic lipid changes, the precise molecular pathways—particularly involving adipokines and mononuclear cell lipid metabolism—require further elucidation [8,13,14,29,36,51].

Long-term cardiovascular outcomes of newer therapies: While biologics and JAK inhibitors modulate lipid profiles, prospective studies are needed to confirm reductions in myocardial infarction, stroke, and atherosclerotic plaque progression [10,23,53].

Optimal integration of lifestyle and pharmacologic interventions: Evidence suggests synergistic benefits, but randomized controlled trials quantifying impact on lipid metabolism and CV events are limited [32,34,56].

Personalized risk stratification: Advanced lipidomics, metabolomics, and imaging tools must be validated in large PsA cohorts to guide therapy selection and optimize outcomes [4,8,21,53].

Potential Study Designs

To address these gaps, future studies should include:

Prospective cohorts assessing lipid profile evolution and cardiovascular events in PsA patients initiating biologics or JAK inhibitors [2,33,52,57].

Randomized trials combining weight loss interventions with DMARD therapy to evaluate additive effects on lipid metabolism and disease activity [32,34,59].

Integrative biomarker studies using adipokine profiling, lipidomics, and imaging to develop predictive models for personalized therapy [4,5,8,17,21].

Pharmacologic adjunct trials of statins, PCSK9 inhibitors, or ApoA1 mimetics in PsA populations at high CV risk [10,50,53].

Translational and Clinical Implications

Advances in understanding PsA-associated dyslipidemia will allow:

Identification of high-risk patients before overt cardiovascular disease develops

Early intervention with combined anti-inflammatory, lipid-modifying, and lifestyle strategies

Optimization of therapeutic sequencing to maximize both joint/skin disease control and cardiometabolic protection [9,32,34,56]

Ultimately, integrating metabolic, inflammatory, and cardiovascular management represents a paradigm shift from symptom control to holistic disease modification in PsA (Table 3).

## 3. Summary

In conclusion, psoriatic arthritis represents a systemic inflammatory condition in which immune-mediated mechanisms, adipokine imbalance, and metabolic dysfunction converge to promote a pro-atherogenic lipid profile and increased cardiovascular risk. The available evidence consistently demonstrates alterations in lipid parameters and adipokine levels; however, their causal relationships with disease activity remain incompletely understood due to heterogeneity across studies.

Importantly, these metabolic disturbances are not merely comorbid findings but appear to be integral components of disease pathophysiology, contributing to both inflammation and long-term cardiovascular outcomes. This highlights the need for early identification and comprehensive management of cardiometabolic risk factors in patients with PsA.

Future research should focus on longitudinal and mechanistic studies to clarify causal pathways and to determine whether targeted metabolic interventions can modify disease progression and cardiovascular risk. A multidisciplinary approach integrating rheumatologic, metabolic, and cardiovascular care remains essential for optimizing patient outcomes.

## Figures and Tables

**Table 1:** Cardiovascular risk markers and interventions in PsA.

Parameter	Finding in PsA	Clinical Implication	Reference
HDL	↓	↑CV risk	[1,2,24]
LDL	↑	↑CV risk	[2,26]
ApoB/ApoA1 ratio	↑	Predictor of atherosclerosis	[2,4,26]
Triglycerides	↑	↑CV risk	[3,9]
Weight loss	↓Disease activity, ↑HDL	Therapeutic strategy	[32,34,56]
TNF inhibitors	Modulate inflammation, improve lipids	Therapeutic strategy	[33,34,52,57]
Statins	↓LDL, stabilize plaques	Adjunctive therapy	[10,23,50]

**Table 2:** Effects of PsA therapies on lipid metabolism and CV risk.

Therapy	Effect on Lipid Profile	Effect on CV Risk	Reference
Methotrexate	↓ApoB/ApoA1, ↑HDL	↓CV risk	[2,24]
TNF inhibitors	↑HDL, ↓LDL/ApoB	↓CV events	[33,34,52,57,66,68]
IL-17/IL-23 inhibitors	↑HDL functionality, ↓CRP	Potential ↓atherosclerosis	[5,10,17,20,23]
Weight loss	↑HDL, ↓leptin, ↓TG	↓CV risk, ↓disease activity	[32,34,56]
Physical activity & diet	↓TG, ↑HDL, improved insulin sensitivity	↓CV risk	[9,43,68]
Statins	↓LDL, plaque stabilization	↓CV events	[10,23,50]

**Table 3:** Future therapeutic strategies and research directions in PsA.

Strategy	Potential Benefit	Current Evidence	Reference
JAK inhibitors	↓Inflammation, ↑HDL, ↓ApoB/ApoA1	Early studies; long-term CV data pending	[10,20,24,26,36]
IL-17/IL-23 inhibitors	↓Vascular inflammation, ↑HDL functionality	Preliminary clinical data	[5,17,20,23]
Lipid-targeted agents (statins, PCSK9, ApoA1 mimetics)	↓LDL, plaque stabilization	Extrapolated from CV studies	[10,50,53]
Weight loss & lifestyle	↑HDL, ↓leptin, ↓TG, improved insulin sensitivity	Interventional studies	[9,32,34,56]
Biomarker-guided therapy	Personalized intervention, improved CV risk stratification	Early validation	[4,5,8,17,21,53]
Integrated multimodal therapy	Synergistic CV and disease activity benefits	Conceptual & pilot studies	[2,32,34,52,56,58]

## Data Availability

No new data were created or analyzed in this study.

## Abbreviations

The following abbreviations are used in this manuscript:

PsA	Psoriatic arthritis
ApoA1	apolipoprotein A1
ApoB	apolipoprotein B
HDL	high-density lipoprotein
LDL	low-density lipoprotein
VLDL	very low-density lipoprotein
TNF inhibitors	Tumor necrosis factor inhibitors
DMARDs	Disease-modifying antirheumatic drugs
cDMARDs	conventional Disease-modifying antirheumatic drugs
TG	triglycerides
CV	cardiovascular
IMT	intima-media thickness
BMI	Body mass index
JAK	Janus kinase
